# Air Pollution and Primary DNA Damage among Zagreb (Croatia) Residents: A Cross-Sectional Study

**DOI:** 10.3390/jox14010023

**Published:** 2024-03-13

**Authors:** Marko Gerić, Gordana Pehnec, Katarina Matković, Jasmina Rinkovec, Ivana Jakovljević, Ranka Godec, Silva Žužul, Ivan Bešlić, Ante Cvitković, Luka Delić, Pascal Wild, Irina Guseva Canu, Nancy B. Hopf, Goran Gajski

**Affiliations:** 1 Division of Toxicology, Institute for Medical Research and Occupational Health, 10000 Zagreb, Croatia; 2 Division of Environmental Hygiene, Institute for Medical Research and Occupational Health, 10000 Zagreb, Croatia; 3 Teaching Institute of Public Health Brod-Posavina County, 35000 Slavonski Brod, Croatia; 4 Faculty of Dental Medicine and Health, J. J. Strossmayer University of Osijek, 31000 Osijek, Croatia; 5 Faculty of Medicine, J. J. Strossmayer University of Osijek, 31000 Osijek, Croatia; 6 Center for Primary Care and Public Health (Unisanté), University of Lausanne, 1011 Lausanne, Switzerland; 7 PW Statistical Consulting, 54520 Laxou, France

**Keywords:** air quality, particulate matter, human population, blood cells, DNA damage, comet assay

## Abstract

More than eight million premature deaths annually can be attributed to air pollution, with 99% of the world’s population residing in areas below recommended air quality standards. Hence, the present study aimed to examine the association between primary DNA damage and air pollution data among 123 participants enrolled between 2011 and 2015 in Zagreb, Croatia. While most measured air pollutants adhered to regulatory limits, benzo[a]pyrene concentrations bound to PM_10_ exceeded them. Factorial analysis narrowed down air pollution data to four exposure factors (particulate matter, two metal factors, and other pollutants). Despite the absence of significant positive associations between modeled air pollution exposure factors and comet assay descriptors (tail length, tail intensity, tail moment, and highly damaged nuclei), the critical health implications of air pollution warrant further investigations, particularly with biomarkers of exposure and different biomarkers of effect in populations facing air pollution exposure.

## 1. Introduction

Over the last few decades, air pollution has been recognized as the foremost environmental risk to human health, posing a significant threat globally [1,2,3,4]. Within the exposome framework, lifelong exposure to air-borne pollutants, even at low concentrations, might have a lasting impact at the molecular level [5]. Air pollution is not a single compound; instead, it is a complex mixture of gases such as NOx, ozone, and SOx, along with particulate matter (PM), which include potentially harmful substances like combustion particles, toxic metals, organic compounds, and acidic components. This complexity adds difficulty to the assessment of both exposure and effects [6,7,8,9,10,11,12]. As per WHO, approximately 99% of the world’s population, mainly in low- and middle-income countries, resides in areas with air quality below recommended standards [13], leading to more than 8.5 million excess deaths annually. The imperative for further studies on the health impacts of air pollution is underscored by its association with various diseases [1,2,14,15,16,17,18,19,20,21,22]. 

The comet assay, a reliable tool for measuring strand breaks and a variety of other DNA lesions in human populations, might also be used for predicting risks of noncommunicable diseases and death [23,24]. It also proves valuable in molecular epidemiology, offering sensitivity to detect increased levels of DNA damage and assess ineffective repair mechanisms. This method, quantifying primary DNA damage that can be transformed into single- and double-strand DNA breaks directly or by DNA repair systems, plays a critical role in understanding the biomolecular events in diseases like cancer and degenerative conditions. With advantages such as quick results from low volumes of blood samples and cost-effectiveness, the comet assay scoring can be semi- or fully automated for slide analysis, further optimizing the precision of obtained results [25,26,27,28,29,30,31,32,33,34]. Additionally, its application in the assessment of DNA damage from frozen blood samples allows for large cohort and retrospective studies [35,36]. 

Many reports, including those from the European Environment Agency’s (EEA), suggest that air pollution components are associated to increased risks for certain cancer sites, resulting in elevated premature deaths and substantial economic costs [37,38,39,40]. Previous attempts to associate the air quality parameters with cytokinesis-block micronucleus assay descriptors, another cancer-predictive method, yielded no apparent link for the Zagreb region during the designated period [41]. In this study, we aim to associate air quality data with alkaline comet assay descriptors using historical data from 2011 to 2015. This involves statistically modeling different outcomes of the alkaline comet assay as a function of measured air quality parameters, adjusted for recorded confounders.

## 2. Results

### 2.1. Population Characteristics

The study group comprised 123 participants (83 women and 40 men), with an average age of 39.8 ± 13.6 years (range 19–77). Participants had similar socio-economic status, education level (high school or university), and a comparable body mass index (BMI) of 24.1 ± 4.0 kg/m^2^. Recruited from the general Croatian population in the same region (the city of Zagreb), none of the participants had undergone antibiotic therapy, medical procedures using ionizing radiation, or had been occupationally exposed to genotoxic agents that might potentially impact results for at least three months before blood sampling. Table 1 provides a more detailed description of the studied population.

### 2.2. The Levels of Primary DNA Damage Assessed by the Alkaline Comet Assay

Using the alkaline comet assay, we successfully detected primary DNA damage in the peripheral blood cells obtained from the participants. The mean values of the comet assay descriptors are summarized in Table 2. The tail length (TL), representing the distance of the furthest migrated DNA loop from the comet’s head center, was on average 14.32 ± 1.40 µm (range 12.04–19.45 µm). Tail intensity (TI), indicating the percentage of DNA present in the comet’s tail, was on average 1.62 ± 0.86% (range 0.51–4.19%). Although the tail moment (TM) has recently been discouraged from use due to the lack of units, it is still found in the literature. The average TM was 0.21 ± 0.12 (range 0.06–0.63). To assess the number of highly damaged nuclei, long-tailed nuclei (LTN) and atypically sized tails (AST) were used, representing the 95th percentile of the most damaged cells. The LTN average number was 4.23 ± 5.80, while, for AST, it was 5.30 ± 4.23.

### 2.3. Air Pollution Exposure

Air pollution data were obtained from an air pollution measuring station located in the courtyard of the Institute for Medical Research and Occupational Health in Zagreb. The measuring station has been part of Zagreb’s air quality monitoring network since the 1960s and boasts a comprehensive historical dataset. For the purpose of this study, mass concentrations of selected pollutants from the period 2011 to 2015 were used. Air pollution exposure was calculated for three different fractions of particulate matter, PM_10_, PM_2.5_, and PM_1_ (particulate matter with an equivalent aerodynamic diameter less than 10 µm, 2.5 µm, and 1 µm, respectively), as well as for chemical species present in PM_10_ fraction. In PM_10_, the following species were determined: organic carbon (OC), elemental carbon (EC), acidic ions sulphates (SO_4_^2−^), nitrates (NO_3_^−^) and chlorides (Cl^−^), metals (Pb, Mn, Cd, As, Ni, Cu, Fe, and Zn), and benzo[a]pyrene (B[a]P). Air pollution exposure was calculated for three different time windows: one day, three days, and seven days before blood sampling; Table 3 presents descriptive statistics of air pollution exposure, including the range of average exposure for selected time windows, and the mean and standard deviation of participants’ exposure.

### 2.4. Influence of Air Pollution on the Alkaline Comet Assay Descriptors

When modeling the different comet outcomes as a function of factor scores corresponding to the respective exposure from the previous day, the last three days, and the last seven days, no statistically significant detrimental effects of the exposure were detected (see Supplementary Tables S1–S5). However, some statistically significant, potentially positive effects of the factor scores were noted for the metal factor scores F2 and F4, particularly concerning the mean exposure measurements of the last three days.

## 3. Discussion

Based on historical data (2011–2015) on air pollution measurement and biomarkers of primary DNA damage, we did not detect genomic DNA in blood with PM_10_, PM_2.5_, and PM_1_ below the national PM limits. Genomic DNA damage was not associated to metals or B[a]P concentrations. The alkaline comet assay data are in line with the MN frequency data from our study group sampled during the same period [41]. 

In terms of air quality for the designated period, we observed that the concentrations of metals Pb, As, Cd, and Ni for the period 2011–2015 were much lower than current limit or target values set for an annual average by Croatian and European Union legislation (0.5 µg/m^3^, 6 ng/m^3^, 5 ng/m^3^, and 20 ng/m^3^ for Pb, As, Cd, and Ni, respectively). Annual concentrations of PM_2.5_ ranged between 19 µg/m^3^ and 25 µg/m^3^ (the limit value is 25 µg/m^3^). Considering PM_10_, annual averages were between 24 µg/m^3^ and 34 µg/m^3^, lower than the limit value set for annual averages (40 µg/m^3^). However, during winter months, daily PM_10_ averages occasionally exceeded the limit value set for a 24 h averaging period (50 µg/m^3^). Annual B[a]P mass concentrations were between 0.8 and 1.3 ng/m^3^ (target value for B[a]P is 1 ng/m^3^ for annual average). This is in accordance with other studies of air quality in Croatia, where PM_10_ exceedances were observed during the winter period, along with relatively high B[a]P mass concentrations [42,43]. These pollutants showed distinct seasonal variations, with significantly higher values during the cold part of the year, due to elevated emissions from heating. The mass concentrations of pollutants determined in this study (period 2011–2015) are consistent with research reporting the seasonal nature of air quality in Croatia [43,44,45,46,47,48]. 

When translating exposure to PM_10_ to health risks, there is an estimated 22% increased risk for lung cancer per every 10 µg/m^3^ increase in PM_10_. [49]. Since the comet assay serves as a potential cancer-predictive biomonitoring tool [23], there was a clear rationale for conducting this study. Several studies examined the impacts of high PAH exposure on children residing near heavy traffic roads or industry and noted higher blood cell comet assay descriptors compared to control populations [50,51,52,53,54], while our results align with the biomonitoring results characterizing a reference site with only occasional PM limit exceedances [55]. Some studies used salivary cells as target cells for the air pollution and did not find an increase in DNA damage in relation to air pollutants in Brescia’s preschool children [56]. In Sarajevo, no differences in primary DNA damage were observed when comparing colder periods with poorer air quality to warmer periods, while buccal micronuclei, as a biomarker of effect, detected changes between seasons [57]. This might be because buccal micronuclei measure DNA damage in the target organ, the aerodigestive tract.

There are also several comparable studies associating air pollution and the induction of primary DNA damage in vitro. Air pollutants bound to PM from Italian cities Brescia and Torino managed to induce DNA damage detectable by the comet assay in mice lung A549 and bronchial epithelial BEAS-2B cells [58,59]. Gábelová et al. [60] and Bełcik et al. [61] demonstrated similar results for central and eastern European cities Wrocław, Prague, Košice, and Sofia, indicating that extractable organic matter or PM_2.5_-bound pollutants could induce DNA damage in A549 and hepatocyte HepG2 cells. Using lung cells A549, DNA-damaging induction was also observed for PM_2.5_-bound pollutants present in the city of Küttuga [62]. Notably, the air quality in cities in these studies was generally poorer compared to Zagreb air quality, with PM_10_ and average B[a]P values often exceeding regulatory daily and annual limits of 50 µg/m^3^ and 1 ng/m^3^, respectively. Another important aspect of toxicity assessment involves exposure conditions and particle size. Most of the positive in vitro results usually stem from high-concentration exposures (e.g., 50 m^3^ of air equivalent), while the size of PM influences its penetrability into the respiratory system [63], affecting bioavailability. The mechanisms of air pollution toxicity are driven by oxidative stress and inflammation, which generate a cancer-promoting environment; however, given that the pollutants’ concentration mostly remained within regulatory limits, we might suspect that xenobiotic metabolism and DNA damage repair systems could minimize the DNA-damaging effects of air pollution in our case [64,65,66,67]. Therefore, it would be beneficial to conduct prospective studies where exposure conditions for the studied population would also be considered. In terms of higher exposure to air pollutants, some studies examined biomarkers of exposure and effects in occupationally exposed populations such as oil refinery workers, bus/taxi drivers, policemen, or garagemen. Exposure to benzene, ultra-fine particles, and PAHs induced an increase in tail intensity and oxidative DNA damage [51,68,69,70,71]. 

The results of the present study should be interpreted considering some possible limits, such as the potentially small and nonrepresentative sample, uncontrolled effect modifiers or confounding, selection bias, historical perspective, as well as missing exact biomarkers of exposure. However, human biomonitoring remains the best approach for evaluating the effects of complex mixtures, providing data for further in vitro and in silico studies, and scientifically based risk assessment [72,73,74,75]. Recent studies suggest that sentinel organisms such as butterflies and plants, sensitive to air pollutants, could serve as early warning signs for human biomonitoring studies [76,77,78,79]. Future prospective studies should include biomarkers of air pollution exposure, human exposure conditions, and assessment of biomarkers of oxidative stress and inflammation, as these events might occur earlier in the exposure–effect scenario, forming a basis for DNA damage induction. Finally, developing models that predict biomarkers of effects in human populations based on air quality data would also be beneficial. 

## 4. Materials and Methods

### 4.1. Study Population

We utilized historical data (biomonitoring studies or different ecogenetic studies intended for the determination of baseline DNA damage levels) spanning the years 2011 to 2015, examining 123 participants in Zagreb, Croatia, who were healthy at the time of blood sampling and had provided informed consent. The questionnaire collected demographic, exposure, and lifestyle data, defining a non-smoker as someone abstaining from smoking for a year prior to sample collection. Family history of cancer was self-reported for close family members, and the study considered the seasonality of air pollutants and sun exposure in volunteers to ensure comparability between colder and warmer periods matching in sex ratio, age, smoking status, and BMI. Additionally, participants were excluded if they had occupational exposure, were exposed to ionizing radiation, or had taken antibiotics within three months before sampling took place.

For performing the comet assay on blood cells, a certified medical technician collected venous blood during morning hours from the participants’ donated venous blood into coded heparin-coated tubes (Becton Dickinson, Franklin Lakes, NJ, USA). The coded samples were kept at +4 °C, avoiding light, and processed within the time frame of 4 h after the blood sampling.

### 4.2. Comet Assay

The comet assay on fresh whole blood samples was performed according to a protocol by Collins et al. [28] with some modifications and according to the latest MIRCA guidelines [80]. A volume of 5 µL of whole blood samples was incorporated into 0.5% low melting point (LMP) agarose and added to precoated 1% and 0.6% normal melting point agarose slides. After adding another 0.5% LMP agarose layer, the slides were placed into the lysis solution (pH 10, 2.5 M NaCl, 100 mM Na_2_EDTA, 10 mM Tris, 1% Triton X-100, 1% sodium sarcosinate, and 10% DMSO) and kept overnight at +4 °C. The next day, slides were transferred to electrophoresis solution (pH 13, 300 mM NaOH and 1 mM Na_2_EDTA) at +4 °C and kept for 20 min, following the electrophoresis at 1 V/cm for another 20 min. After the neutralization, slides were dyed with ethidium bromide (10 µg/mL) and analyzed using the Comet assay II program (Perceptive Instruments, Haverhill, UK) connected to the epifluorescence microscope (Leitz, Göttingen, Germany). Comet assay descriptors were calculated using 100 analyzed nucleoids per coded slide in duplicate (200 in total per person). The samples were scored with comet assay upon arrival at the laboratory between 2011 and 2015 (not stored or frozen before scoring).

### 4.3. Air Pollution Measurements

#### 4.3.1. PM Sampling and Gravimetric Measurements

The measuring site for air quality monitoring was located in the northern, residential part of Zagreb, Croatia (45°50′6.8″ N, 15°58′42.12″ E, 168 m a.s.l.), at about 30 m distance from the street, with a modest traffic density. Since the 1960s, the measuring station has been part of the local air-quality-monitoring network funded by the City of Zagreb. It is the only station with continuous parallel measurements of PM_10_, PM_2.5_, and PM_1.0_ particle fractions, as well as detailed PM_10_ chemical content. Considering PM trends at other air-quality-monitoring stations in Zagreb and the absence of similar datasets at other locations, the existing data were considered sufficiently representative for the participants included in the study by Gajski et al. [41].

Daily samples of PM_10_, PM_2.5_, and PM_1.0_ particle fractions were collected every 24 h over a 5-year period, from 2011 to 2015. Low-volume samplers (LVS 3, Sven Leckel Ingenieurbüro GmbH, Berlin, Germany) were used for collecting 55 m^3^ of air/day. Multiple samplers were used to collect PM_10_ on different filter media, depending on the later chemical analysis.

Mass concentrations were determined gravimetrically following the requirements of EN 12341:2066 and EN 14907:2005 standards. A Mettler Toledo MX-5 microbalance (Greifensee, Switzerland) was used for weighing unexposed and exposed filters. Before sampling, filters were weighed twice, the first time after 48 h conditioning at 21 ± 1 °C temperature and 50 ± 5% relative humidity and a second time after 24 h. The mean value of the two weights represents the unexposed filter mass. The same conditioning and weighing procedures were applied to filters after sampling.

#### 4.3.2. Analysis of PM_10_ Content

After sampling and gravimetric determination of PM mass concentrations, in PM_10_ samples, the following constituents were determined: OC, EC, water-soluble anions (SO_4_^2^, NO_3_^−^, and Cl^−^), metals (Pb, Ni, Cd, As, Fe, Cu, Zn, and Mn), and B[a]P.

The thermo-optical transmittance method (TOT) and Quartz (NIOSH-like) protocol were used to determine EC and OC in the PM_10_ fraction on a Carbon Aerosol Analyzer (Sunset Laboratory Inc., Amsterdam, The Netherlands) instrument with a flame ionization detector (FID). QA/QC was ensured with the inner standard, an external sucrose aqueous solution, a check filter, and a cross-method procedure. All other details have been described earlier [41,43].

For the determination of anions (Cl^−^, NO_3_^−^, and SO_4_^2−^), PM_10_ samples were extracted with ultrapure water (18 Milli-Q, resistivity ≥ 18.3 MΩcm) in an ultrasonic bath. The analysis was carried out by ion chromatography on a Dionex DX-120 instrument equipped with a suppressed conductivity detector. Anions were separated on Dionex AS14: 4 mm Analytical Column using AG14: 4 mm Guard Column. The eluent was 3.5 mM Na_2_CO_3_/1 mM NaHCO_3_ solution [41]. 

For the analysis of metals, samples of particulate matter were prepared in a high-pressure microwave digestion system (Ultraclave IV, Milestone, Sorisole, IT) with nitric acid (25% HNO_3_ *v*/*v*) and diluted with deionized water. Thus, digested samples were analyzed using inductively coupled plasma mass spectrometry, ICP-MS (7500cx, Agilent Technologies, Santa Clara, CA, USA). Isotopes ^55^Mn, ^56^Fe, ^60^Ni, ^65^Cu, ^66^Zn, ^75^As, ^111^Cd, and ^206^Pb were selected, and the integration time per point was 0.5 s for As and Cd and 0.1 s for other analyzed metals, with three acquisition points per peak. Scandium, germanium, rhodium, and bismuth were added as internal standards. The ICP-MS spectrometer was tuned to minimize the interferences and maximize the sensitivity and to obtain an oxide ratio and doubly charged ratio < 1.5%. The analysis was made in helium (He) mode. The calibration was carried out every time before sample analysis with working standards (5% HNO3 *v*/*v*) prepared from single-element stock solutions (1000 μg/mL, SCP SCIENCE) at eight-level concentrations. The accuracy of the method was determined by preparing and analyzing reference materials NIST 1648a and ERM CZ120 (PM10-like) in the same way as the collected samples. For both certified reference materials, the recoveries for analyzed metals ranged from 87% to 108% [81].

Extraction of B[a]P from PM_10_ particle fraction was performed in an ultrasonic bath with a solvent mixture of toluene and cyclohexene (7:3, *v*/*v*). Undissolved parts of particles and filter were separated by centrifugation and then evaporated to dryness and redissolved in acetonitrile [82]. B[a]P mass concentration was determined by Varian Pro Star high-performance liquid chromatography (HPLC, Varian, Victoria, Australia) with a fluorescence detector and programed changes in excitation and emission wavelength (λ_excitation_ = 234; λ_emission_ = 500). A mixture of acetonitrile and water was used as a mobile phase with a flow rate of 0.55 mL min^−1^. Data quality assurance and control were achieved by analyzing certified reference material (CRM NIST 1649b, Urban dust) and certificate standard solution (Supelco EPA 610 PAH mix) [42,46]. 

### 4.4. Data Processing and Statistical Analysis

The comet data outcomes were initially merged with the individual subject characteristics and modeled as a function of the latter in order to identify the relevant potential confounders. Multiple linear regression models were used for log_10_-transformed continuous quantitative outcomes (tail length, tail intensity, and tail moments), while multiple Poisson regression models were applied for count responses (LTN and AST). Independent variables with a *p*-value for inclusion below 0.1 were retained. In the second stage, the outcomes were merged with the exposure data. The exposure measurement of the day previous to the blood collection, the mean of the exposure measurements of three previous days, as well as over the seven previous days were considered, respectively, as independent variables. However, some subjects donated the blood samples on the same days for which the exposure measurements were, therefore, identical. The induced correlation was taken into account by incorporating the measurement day as a random effect in the multiple models, which were, therefore, linear mixed models and mixed Poisson models. Given the number of measured exposure variables for which (except for B[a]P) no a priori hypothesis as to their effect on the outcomes was available, the exposure variables were summarized using a factor analysis on log-transformed exposure measurements (details can be found in Gajski et al. [41]). Briefly, four factor scores were generated: a PM factor denoted F1, 2 metal factors (F2—Mn, Cu, Fe, and Z, and F4—Pb, Cd, As, and Zn), and the last factor F3 positively correlated with NO_3_^−^, Cl^−^, OC, EC, and B[a]P—negatively correlated with SO_4_^2−^. Factors 2, 3, and 4 were obtained on concentrations normalized on PM_10_. For each outcome and each time window (previous day, three previous days, and seven previous days), three models were applied; the first included only the PM factor, the second model the PM factor and the two metal factors, whereas the third model included the PM factor and factor F3, characterizing the exposure to other chemicals. 

## 5. Conclusions

Based on our historical data analysis and study’s limits, we can conclude that air pollutants in the period 2011–2015 were not associated with the observed DNA damage levels using alkaline comet assay in this study. Most of the air quality data remained within the regulatory limits. Given the transboundary nature of air pollution, our results might be regionally important. Since air pollution is considered a significant health issue, and air pollution is often site-specific, more studies using biomarkers of exposure, as well as using different biomarkers of effect could be expected. This approach would also contribute to the development of appropriate models for the prediction of air-pollution-induced effects on the human population.

## Figures and Tables

**Table 1:** Main characteristics and lifestyle factors of the study population.

	Total	Women	Men
N	123	83	40
Age (years)	39.8 ± 13.6	40.2 ± 14.3	38.9 ± 12.0
Age range (years)	19–77	19–77	24–64
BMI (kg/m^2^)	24.1 ± 4.0	23.4 ± 4.1	25.7 ± 3.4
Current smokers (%)	28.5	30.1	25.0
Family history of cancer (%)	41.5	50.6	22.5

BMI, body mass index; N, number of volunteers. Indicated as the number or average ± standard deviation.

**Table 2:** Mean values and standard deviation of the comet assay outcomes measured in peripheral blood cells of the studied population.

	Total	Women	Men
**Tail length (µm)**	14.32 ± 1.40	14.30 ± 1.50	14.35 ± 1.18
**[range]**	[12.04–19.45]	[12.04–19.45]	[12.69–16.74]
**Tail intensity (%)**	1.62 ± 0.86	1.57 ± 0.82	1.74 ± 0.93
**[range]**	[0.51–4.19]	[0.51–4.09]	[0.57–4.19]
**Tail moment**	0.21 ± 0.12	0.21 ± 0.12	0.23 ± 0.13
**[range]**	[0.06–0.63]	[0.06–0.63]	[0.07–0.57]
**LTN**	4.23 ± 5.80	4.46 ± 6.55	3.75 ± 3.81
**[range]**	[0–33.5]	[0–33.5]	[0–16.5]
**AST**	5.30 ± 4.23	4.95 ± 3.87	6.04 ± 4.87
**[range]**	[0–20.5]	[0–15.5]	[0–20.5]

AST, atypically sized tails—number of nuclei exceeding 95th percentile of tail intensity; LTN, long-tailed nuclei—number of nuclei exceeding 95th percentile of tail length.

**Table 3:** Air pollution exposure of study participants for different time windows (one, three, and seven days before blood sampling).

Pollutant	Average 1 Day Before		Average 3 Days Before		Average 7 Days Before	
	Mean (SD)	Range	Mean (SD)	Range	Mean (SD)	Range
**PM_10_ (µg/m^3^)**	31 (17)	5–88	29 (15)	9–79	29 (13)	12–67
**PM_2.5_ (µg/m^3^)**	24 (16)	3–73	23 (14)	6–68	22 (12)	7–59
**PM_1_ (µg/m^3^)**	17 (10)	1–44	16 (9)	6–39	16 (8)	7–37
**OC (µg/m^3^)**	8.29 (4.70)	1.91–18.54	7.87 (4.23)	3.02–18.73	7.85 (3.90)	3.50–18.20
**EC (µg/m^3^)**	1.05 (0.55)	0.24–2.62	1.03 (0.57)	0.31–2.94	1.13 (0.59)	0.42–3.57
**SO_4_^2−^ (µg/m^3^)**	5.49 (7.27)	1.03–40.63	4.45 (5.24)	1.07–31.30	4.11 (3.47)	1.17–19.88
**NO_3_^−^ (µg/m^3^)**	3.15 (3.52)	0.04–15.96	3.21 (3.28)	0.25–12.15	3.39 (3.19)	0.34–16.10
**Cl^−^ (µg/m^3^)**	0.19 (0.31)	0.01–1.30	0.19 (0.29)	0.01–1.42	0.22 (0.31)	0.01–1.44
**Pb (µg/m^3^)**	0.007 (0.005)	0.001–0.034	0.006 (0.004)	0.002–0.017	0.006 (0.003)	0.002–0.016
**Mn (µg/m^3^)**	0.005 (0.002)	0.002–0.012	0.005 (0.002)	0.001–0.012	0.006 (0.002)	0.002–0.010
**Cd (ng/m^3^)**	0.270 (0.293)	0.041–1.698	0.215 (0.169)	0.025–0.884	0.217 (0.163)	0.039–0.837
**As (ng/m^3^)**	0.649 (0.603)	0.077–3.162	0.532 (0.428)	0.132–2.118	0.524 (0.316)	0.179–1.640
**Ni (ng/m^3^)**	1.139 (1.888)	LOD–10.479	1.004 (1.082)	LOD–5.018	1.044 (0.973)	LOD–5.454
**Cu (µg/m^3^)**	0.012 (0.007)	0.003–0.033	0.012 (0.006)	0.003–0.034	0.013 (0.005)	0.004–0.025
**Fe (µg/m^3^)**	0.297 (0.168)	0.076–0.775	0.295 (0.160)	0.073–0.958	0.318 (0.126)	0.092–0.712
**Zn (µg/m^3^)**	0.023 (0.014)	0.005–0.071	0.020 (0.010)	0.004–0.057	0.021 (0.009)	0.005–0.055
**B[a]P (ng/m^3^)**	1.035 (1.577)	LOD–9.541	1.049 (1.233)	0.029–5.307	0.988 (1.037)	0.039–3.517

B[a]P—benzo[a]pyrene, EC—elemental carbon, LOD—limit of detection, OC—organic carbon, SD—standard deviation.

## Data Availability

The original contributions generated for this study are included in the article. Further inquiries can be directed to the corresponding author upon reasonable request.

## Supplementary Materials

The following supporting information can be downloaded at: https://www.mdpi.com/article/10.3390/jox14010023/s1. Table S1. Mean tail length. Table S2. Mean tail intensity. Table S3. Mean tail moment. Table S4. LTN numbers tail length above 95%. Table S5. AST numbers intensity above 95%.
