# Supplementary material for: Air Pollution and Primary DNA Damage among Zagreb (Croatia) Residents: A Cross-Sectional Study
Source: J Xenobiot. 2024 Mar 13;14(1):368–79. doi: 10.3390/jox14010023 (PMC10971122; doi:10.3390/jox14010023)
Supplement: Supplementary file 1 [file jox-14-00023-s001.zip › jox-2874009-supplementary.pdf]

Air Pollution and Primary DNA Damage among Zagreb (Croatia) Residents: A Cross-Sectional Study

Table S1. Mean tail length.

	Previous day			3 previous days			7 previous days		
	PM	PM+ metals	PM+ other	PM	PM+ metals	PM+ other	PM	PM+ metals	PM+ other
Temperature									
<2°	-	-	-	-	-	-	-	-	-
2°-<10°									
Coefficient	0.004	0.005	0.004	0.012	0.011	0.009	0.010	0.008	0.007
p-value	0.816	0.761	0.807	0.506	0.528	0.585	0.570	0.733	0.669
10°-<18°									
Coefficient	0.022	0.031	0.021	0.032	0.040	0.041	0.031	0.028	0.037
p-value	0.228	0.160	0.260	0.064	0.041	0.024	0.077	0.234	0.050
>18°									
Coefficient	0.026	0.030	0.024	0.043	0.041	0.063	0.040	0.038	0.052
p-value	0.195	0.231	0.294	0.052	0.092	0.016	0.066	0.175	0.047
F1									
Coefficient	0.002	0.009	-0.004	0.037	0.061	0.047	0.035	0.033	0.037
p-value	0.939	0.729	0.901	0.264	0.067	0.160	0.340	0.385	0.316
F2									
Coefficient		-0.035			-0.050			0.010	
p-value		0.161			0.088			0.805	
F4									
Coefficient		-0.046			-0.109			0.010	
p-value		0.146			0.018			0.864	
F3									
Coefficient			-0.003			0.035			0.026
p-value			0.885			0.172			0.413

Linear mixed models (one model per column) of the log10 transformed mean tail length as a function of factor scores of the exposure measurements adjusted on the temperature.

Table S2. .Mean tail intensity

	Previous day			3 previous days			7 previous days		
	PM	PM+ metals	PM+ other	PM	PM+ metals	PM+ other	PM	PM+ metals	PM+ other
Temperature									
<2°	-	-	-	-	-	-	-	-	-
2°-<10°									
Coefficient	-0.066	-0.032	-0.053	-0.034	-0.011	-0.028	-0.032	0.000	-0.017
p-value	0.460	0.723	0.541	0.710	0.901	0.756	0.717	0.997	0.853
10°-<18°									
Coefficient	0.036	0.155	-0.007	0.088	0.191	0.076	0.086	0.142	0.060
p-value	0.715	0.194	0.946	0.357	0.066	0.456	0.374	0.272	0.561
>18°									
Coefficient	0.118	0.231	0.019	0.196	0.248	0.170	0.187	0.224	0.128
p-value	0.271	0.084	0.873	0.107	0.054	0.247	0.115	0.140	0.376
F1									
Coefficient	-0.098	-0.009	-0.191	0.055	0.216	0.030	0.079	0.125	0.066
p-value	0.475	0.950	0.182	0.751	0.203	0.868	0.681	0.523	0.728
F2									
Coefficient		-0.256			-0.411			-0.180	
p-value		0.043			0.006			0.382	
F4									
Coefficient		-0.116			-0.556			-0.244	
p-value		0.473			0.017			0.424	
F3									
Coefficient			-0.183			-0.089			-0.175
p-value			0.079			0.511			0.278

Linear mixed models (one model per column) of the log10 transformed mean tail intensity as a function of factor scores of the exposure measurements adjusted on the temperature.

Table S3. Mean tail moment

	Previous day			3 previous days			7 previous days		
	PM	PM+ metals	PM+ other	PM	PM+ metals	PM+ other	PM	PM+ metals	PM+ other
Temperature									
<2°	-	-	-	-	-	-	-	-	-
2°-<10°									
Coefficient	-0.039	0.000	-0.027	-0.003	0.023	-0.000	-0.008	0.032	0.003
p-value	0.676	0.996	0.771	0.976	0.802	1.000	0.929	0.790	0.977
10°-<18°									
Coefficient	0.036	0.155	-0.007	0.088	0.191	0.076	0.086	0.142	0.060
p-value	0.715	0.194	0.946	0.357	0.066	0.456	0.374	0.272	0.561
>18°									
Coefficient	0.118	0.231	0.019	0.196	0.248	0.170	0.187	0.224	0.128
p-value	0.271	0.084	0.873	0.107	0.054	0.247	0.115	0.140	0.376
F1									
Coefficient	-0.053	0.045	-0.145	0.126	0.295	0.112	0.123	0.165	0.066
p-value	0.713	0.760	0.342	0.494	0.100	0.550	0.543	0.424	0.728
F2									
Coefficient		-0.280			-0.430			-0.178	
p-value		0.036			0.006			0.411	
F4									
Coefficient		-0.118			-0.580			-0.185	
p-value		0.491			0.019			0.566	
F3									
Coefficient			-0.178			-0.046			-0.122
p-value			0.106			0.747			0.477

Linear mixed models (one model per column) of the log10 transformed mean tail moment as a function of factor scores of the exposure measurements adjusted on the temperature.

Table S4. LTN numbers tail length above 95%

	Previous day			3 previous days			7 previous days		
	PM	PM+ metals	PM+ other	PM	PM+ metals	PM+ other	PM	PM+ metals	PM+ other
Sampling Year									
2011	-	-	-	-	-	-	-	-	-
2012									
Coefficient	-0.113	-0.059	0.101	-0.363	-0.484	-0.319	-0.095	-0.414	0.016
p-value	0.852	0.922	0.866	0.562	0.422	0.626	0.872	0.481	0.979
2013									
Coefficient	0.576	0.364	0.973	0.577	0.247	0.672	0.536	0.618	0.761
p-value	0.368	0.593	0.140	0.358	0.692	0.371	0.398	0.333	0.278
2014									
Coefficient	-0.905	-0.801	-0.739	-0.924	-0.774	-0.905	-0.867	-1.114	-0.806
p-value	0.002	0.006	0.015	0.002	0.006	0.003	0.004	0.000	0.009
2015									
Coefficient	0.795	0.874	1.415	0.744	0.836	0.849	0.843	0.894	1.169
p-value	0.105	0.067	0.018	0.126	0.069	0.204	0.086	0.054	0.078
Temperature									
<2°	-	-	-	-	-	-	-	-	-
2°-<10°									
Coefficient	0.218	0.267	0.235	0.413	0.433	0.417	0.278	1.042	0.341
p-value	0.640	0.565	0.601	0.380	0.341	0.376	0.540	0.075	0.457
10°-<18°									
Coefficient	1.239	1.615	1.057	1.461	1.773	1.419	1.352	2.112	1.249
p-value	0.012	0.005	0.030	0.002	0.001	0.005	0.004	0.001	0.011
>18°									
Coefficient	1.364	1.683	0.886	1.782	1.881	1.678	1.579	2.583	1.290
p-value	0.008	0.010	0.118	0.002	0.003	0.022	0.004	0.000	0.056
F1									
Coefficient	0.187	0.471	-0.331	1.139	1.776	1.064	0.789	0.600	0.789
p-value	0.772	0.470	0.632	0.192	0.039	0.252	0.389	0.502	0.386
F2									
Coefficient		-1.153			-1.691			-0.844	
p-value		0.057			0.020			0.401	
F4									
Coefficient		-0.701			-2.570			2.832	
p-value		0.420			0.037			0.064	
F3									
Coefficient			-1.077			-0.209			-0.764
p-value			0.086			0.818			0.468

Mixed Poisson models (one model per column) of the number of cells above the 95th percentile of the tail length (LTN) as a function of factor scores of the exposure measurements adjusted on the temperature and the sampling year.

Table S5. AST numbers intensity above 95%

	Previous day			3 previous days			7 previous days		
	PM	PM+ metals	PM+ other	PM	PM+ metals	PM+ other	PM	PM+ metals	PM+ other
Gender									
Female	-	-	-	-	-	-	-	-	-
Male									
Coefficient	0.155	0.164	0.151	0.159	0.175	0.159	0.160	0.159	0.160
p-value	0.049	0.037	0.055	0.043	0.026	0.043	0.042	0.044	0.042
Body mass index									
Healthy	-	-	-	-	-	-	-	-	-
Overweight									
Coefficient	-0.028	-0.027	-0.026	-0.029	-0.034	-0.029	-0.029	-0.030	-0.029
p-value	0.741	0.754	0.761	0.738	0.692	0.735	0.732	0.724	0.735
Obese									
Coefficient	-0.145	-0.158	-0.135	-0.149	-0.156	-0.151	-0.146	-0.147	-0.144
p-value	0.319	0.276	0.356	0.308	0.281	0.304	0.315	0.313	0.327
Temperature									
<2°	-	-	-	-	-	-	-	-	-
2°-<10°									
Coefficient	-0.270	-0.115	-0.234	-0.178	0.004	-0.184	-0.112	-0.178	-0.101
p-value	0.409	0.720	0.473	0.602	0.991	0.593	0.733	0.702	0.764
10°-<18°									
Coefficient	-0.090	0.389	-0.188	0.060	0.512	0.080	0.173	0.112	0.150
p-value	0.793	0.336	0.599	0.860	0.184	0.831	0.612	0.824	0.686
>18°									
Coefficient	0.286	0.744	0.083	0.469	0.810	0.508	0.634	0.545	0.587
p-value	0.430	0.095	0.844	0.276	0.085	0.334	0.119	0.334	0.247
F1									
Coefficient	-0.292	0.066	-0.477	0.142	0.809	0.162	0.603	0.601	0.597
p-value	0.538	0.888	0.349	0.829	0.220	0.811	0.390	0.411	0.395
F2									
Coefficient		-1.122			-1.489			0.068	
p-value		0.013			0.009			0.936	
F4									
Coefficient		-0.389			-1.498			-0.270	
p-value		0.511			0.084			0.808	
F3									
Coefficient			-0.371			0.068			-0.097
p-value			0.352			0.897			0.876

Mixed Poisson models (one model per column) of the number of cells above the 95th percentile of the tail intensity (AST) as a function of factor scores of the exposure measurements adjusted on the temperature, gender and BMI (in three categories)
